# Complete Resection of a Middle Mediastinal Malignant Peripheral Nerve Sheath Tumour via a Transmanubrial Osteomuscular Sparing Approach: A Case Report

**DOI:** 10.1002/rcr2.70664

**Published:** 2026-07-02

**Authors:** Minoru Sugihara, Shota Nakamura, Heng Huang, Taiki Ryo, Yoshito Imamura, Yasuhisa Ichikawa, Tatsuya Masuda, Hirofumi Takenaka, Hiroki Watanabe, Yuta Kawasumi, Keita Nakanishi, Yuka Kadomatsu, Harushi Ueno, Taketo Kato, Tetsuya Mizuno, Toyofumi Fengshi Chen‐Yoshikawa

**Affiliations:** ^1^ Department of Thoracic Surgery Nagoya University Graduate School of Medicine Nagoya Aichi Japan

**Keywords:** malignant peripheral nerve sheath tumour, middle mediastinum, transmanubrial osteomuscular sparing approach

## Abstract

Malignant peripheral nerve sheath tumour (MPNST) is a rare soft‐tissue sarcoma, and mediastinal involvement—particularly in the middle mediastinum—is exceedingly uncommon. Complete resection is crucial for favourable outcomes, as MPNST is generally resistant to chemotherapy and radiotherapy. We report a case of a middle mediastinal MPNST completely resected using a transmanubrial osteomuscular sparing approach (TMA). A 35‐year‐old man with neurofibromatosis type 1 presented with an enlarging 6.8‐cm middle mediastinal mass suspected to be a malignant neurogenic tumour. Although initially considered unresectable due to its proximity to major mediastinal structures, surgical exploration using a TMA enabled complete resection. The tumour originated from the right vagus nerve, and histopathological examination confirmed MPNST with negative margins. This case highlights the importance of not deeming tumours unresectable based solely on preoperative imaging, but rather evaluating the potential for complete resection through intraoperative findings in selected cases.

## Graphical abstract

We report a rare middle mediastinal malignant peripheral nerve sheath tumour. Preoperative imaging showed contact with vital structures, raising concern for resectability. However, complete resection was achieved using a transmanubrial osteomuscular sparing approach.

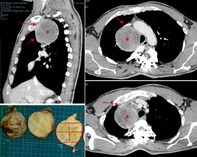

## 1 Introduction

Malignant peripheral nerve sheath tumour (MPNST) is a rare soft‐tissue sarcoma. Its occurrence in the mediastinum is uncommon and is reported predominantly in the posterior mediastinum [1, 2]. MPNST is generally resistant to chemotherapy and radiotherapy; however, favourable outcomes have been reported after complete surgical resection [1, 2].

Herein, we report a case of a middle mediastinal MPNST in which preoperative imaging indicated contact with vital structures and considerable technical difficulty, thereby casting doubt on resectability. Employing a transmanubrial osteomuscular sparing approach (TMA), complete resection was accomplished, indicating that careful surgical planning and appropriate assessment can expand the boundaries of resectability in selected mediastinal tumours.

## 2 Case Report

The patient was a 35‐year‐old man with neurofibromatosis type 1 (NF1) and a history of scoliosis treated with spinal fusion surgery. Follow‐up chest computed tomography (CT) detected an enlarging middle mediastinal mass. Transbronchial biopsy revealed a malignant neurogenic tumour.

**FIGURE 1:**
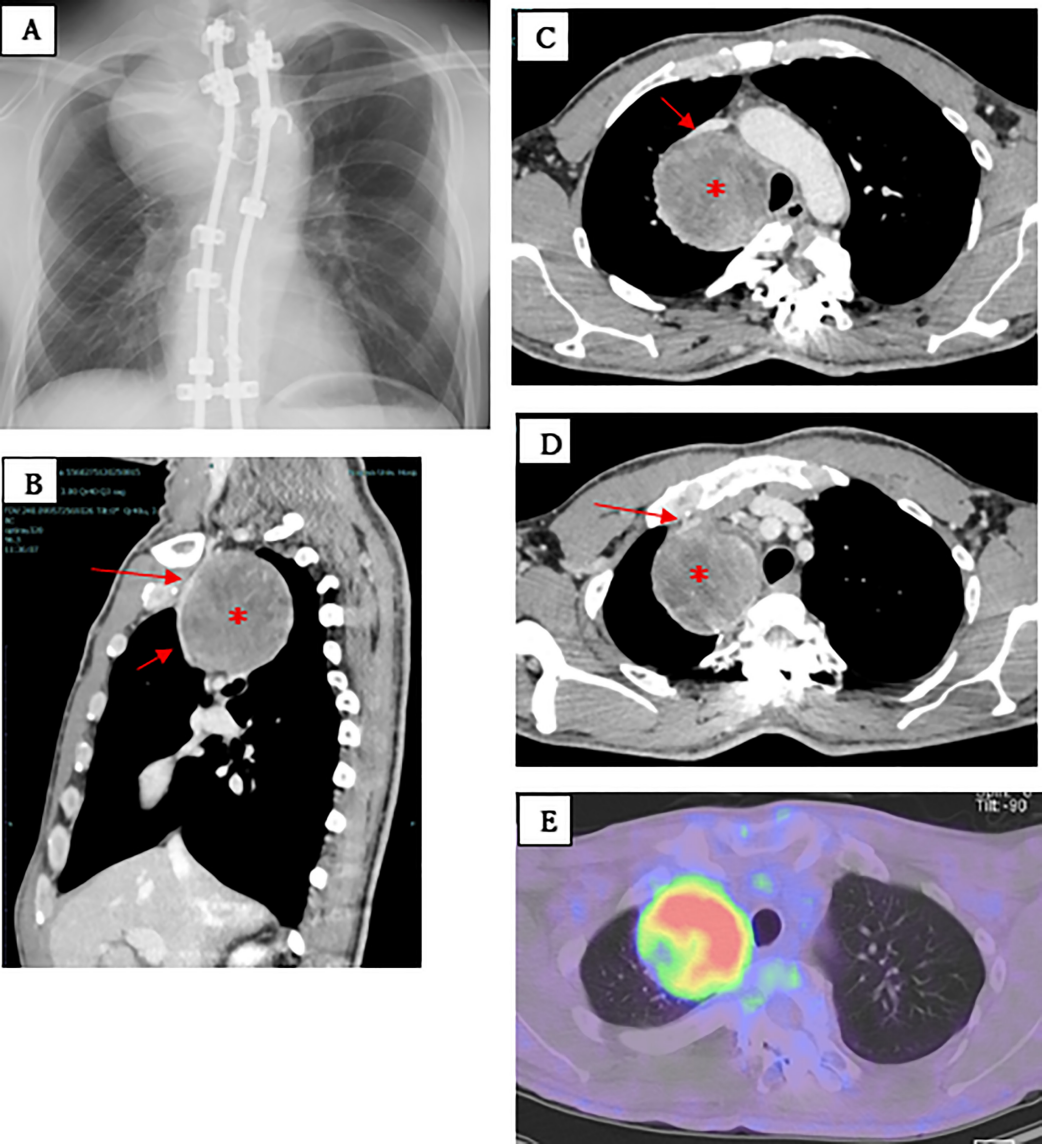
Imaging findings. Chest radiography showed a mass in the right upper to middle lung fields with tracheal deviation (A). CT demonstrated a mass (asterisk) in the right middle mediastinum compressing the SVC (short arrow) and right brachiocephalic vein (long arrow) (B–D). PET–CT showed FDG uptake (SUVmax, 8.5) (E).

Chest radiography revealed a mass in the right upper to middle thoracic region, with leftward tracheal displacement (Figure 1A). CT demonstrated a heterogeneously enhanced 6.8‐cm mass in the right middle mediastinum, leading to leftward tracheal deviation and compressing the superior vena cava (SVC) and right brachiocephalic vein (Figure 1B–D). Positron emission tomography (PET)–CT revealed fluorodeoxyglucose uptake with a maximum standardized uptake value (SUVmax) of 8.5 (Figure 1E).

Grounded on these findings, the tumour was diagnosed as a middle mediastinal MPNST. The tumour was in extensive contact with adjacent vital structures, including the trachea, lung, SVC, and right brachiocephalic vein. This raised substantial concern regarding both the technical feasibility of safe dissection and potential invasion into major organs and vessels. During multidisciplinary tumour board discussion, the tumour was initially regarded as unresectable by several thoracic surgeons, and nonsurgical treatment was recommended; however, the anticipated prognosis was deemed unfavourable. Considering that complete surgical resection represents the most important determinant of long‐term survival in MPNST, we decided to reassess resectability intraoperatively and to pursue complete resection whenever technically feasible. Thoracoscopic exploration alone was insufficient to assess tumour invasion or dissection safety; thus, a TMA was selected as the initial surgical strategy.

We planned to add a Trap‐door or hemi‐clamshell approach to enable combined resection of the involved organs and major vessels and vascular reconstruction if intraoperative findings revealed invasion into adjacent organs or major vessels, or if tumour dissection proved technically difficult. The surgical strategy was discussed with cardiac surgeons in anticipation of possible cardiopulmonary bypass and major vascular reconstruction.

**FIGURE 2:**
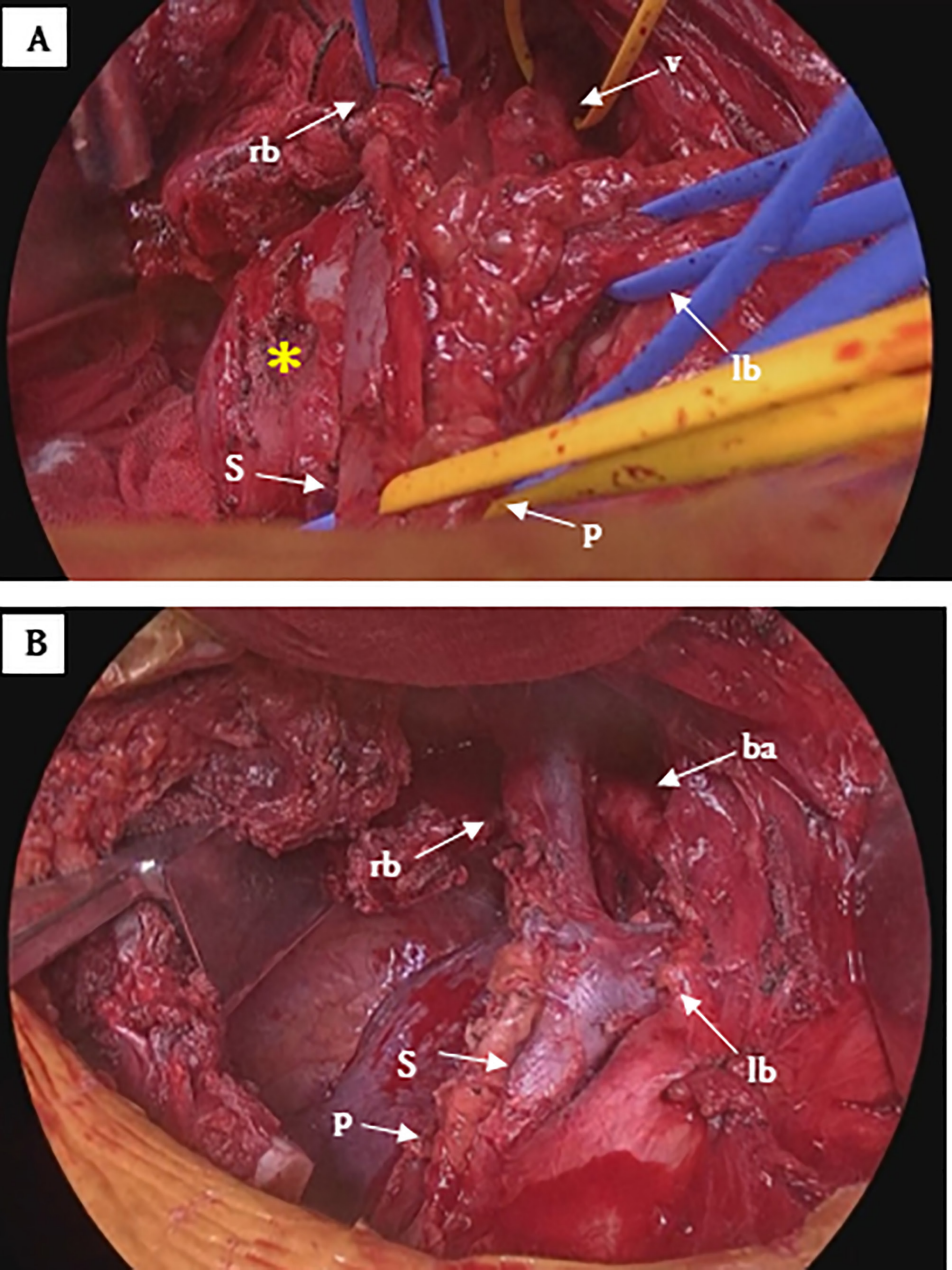
Surgical findings. The tumour (asterisk) was identified within the mediastinal pleura (A). The tumour was completely resected as a mass continuous with the right vagus nerve (B). ba: brachiocephalic artery; lb: left brachiocephalic vein; p: phrenic nerve; rb: right brachiocephalic vein; S: SVC; v: vagus nerve.

The patient underwent a TMA in the supine position, which involved division of the first and second ribs and an inverted L‐shaped sternal incision at the second intercostal space. A soft, elastic mass approximately 6.5 cm in size was identified within the mediastinal pleura, with no obvious invasion into adjacent organs or major vessels. The tumour originated from the right vagus nerve, which was carefully dissected from the trachea, bilateral brachiocephalic veins, brachiocephalic artery, SVC, and right phrenic nerve, and was completely resected with part of the right vagus nerve. The divided manubrium was reapproximated using stainless steel wires (Figure 2A,B, Video 1).

**VIDEO 1:** Intraoperative video demonstrating complete resection of an MPNST via a TMA. The tumour was carefully dissected from the surrounding structures and resected with part of the right vagus nerve. Video content can be viewed at https://onlinelibrary.wiley.com/doi/10.1002/rcr2.70664.

**FIGURE 3:**
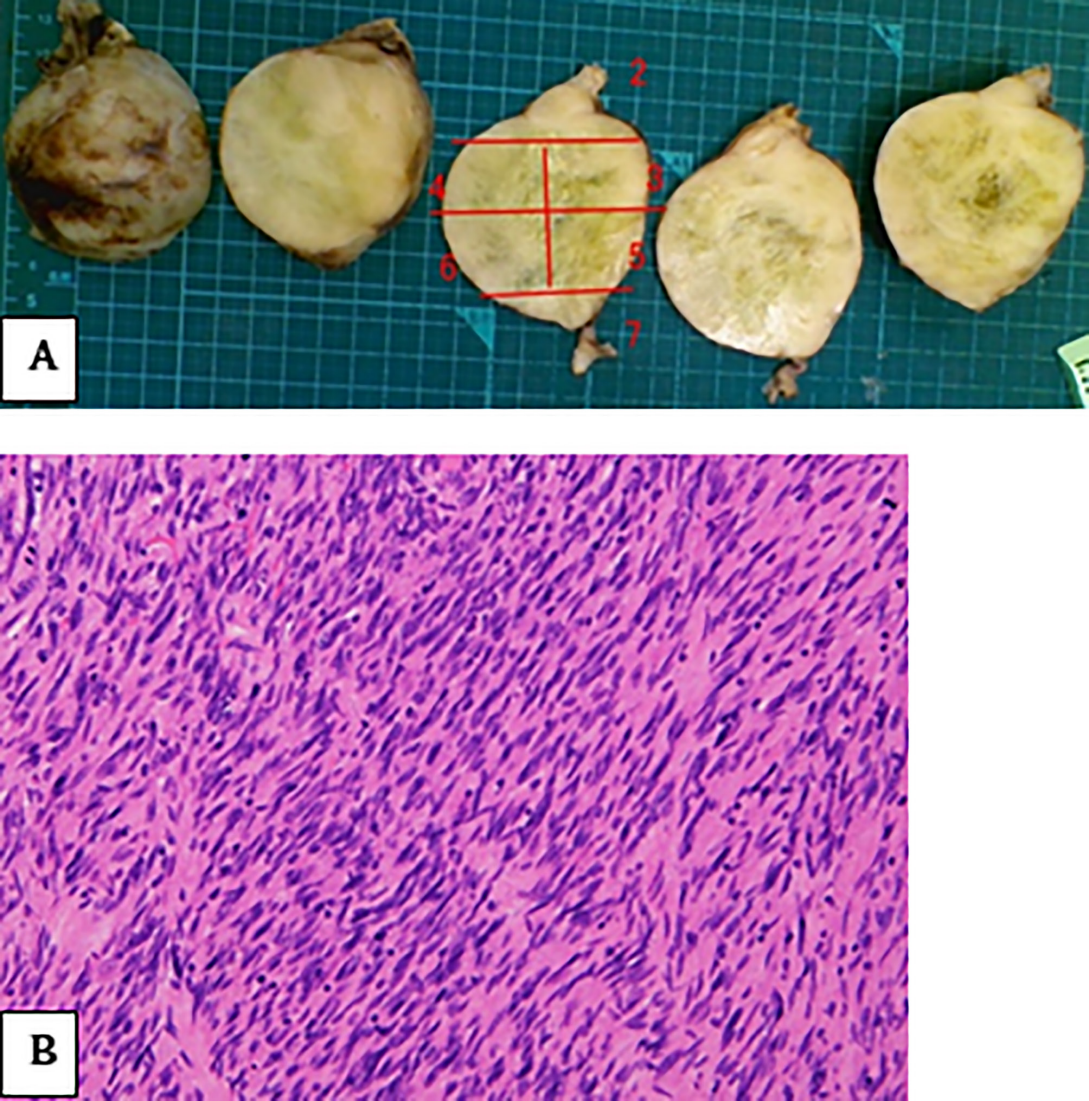
Pathological findings. The specimen was well‐circumscribed with a whitish cut surface (A). Histopathology showed spindle‐shaped tumour cells (B).

Postoperatively, the patient developed right recurrent laryngeal nerve palsy. However, after swallowing rehabilitation, he resumed oral intake without difficulty and was discharged on postoperative day 11. The resected tumour was a well‐circumscribed mass with a whitish cut surface (Figure 3A). Histopathological examination revealed spindle‐shaped tumour cells arranged in fascicular and interlacing patterns (Figure 3B). Immunohistochemically, the tumour cells were positive for S‐100 protein and SOX10, and the Ki‐67 labelling index was high (20%–30%), consistent with MPNST. All margins were negative, thereby confirming complete resection.

## 3 Discussion

MPNST is a rare soft‐tissue sarcoma, accounting for approximately 5% of malignant soft‐tissue tumours. It most commonly originates from the extremities and trunk [2], whereas mediastinal occurrence is rare and predominantly involves the posterior mediastinum [1]. Unlike superficially located MPNSTs, mediastinal MPNSTs frequently remain asymptomatic for longer periods and are commonly unresectable at diagnosis [1]. Complete surgical resection is a crucial prognostic factor in MPNST, whereas chemotherapy or radiotherapy has limited efficacy in unresectable cases, leading to poor prognosis [1]. Poor prognostic factors for MPNST include younger age (≤ 30 years), large tumour size (≥ 5 cm), high grade, truncal location, NF1, and incomplete resection [1, 2].

The present case involved an MPNST originating from the middle mediastinum—an atypical location. To date, only three cases of completely resected middle mediastinal MPNST have been reported. The reported surgical approaches included median sternotomy and posterolateral thoracotomy, whereas the approach was not described in one case [1, 3, 4]. For middle mediastinal MPNST with anticipated invasion into adjacent organs or technical difficulty in tumour dissection, the TMA represents a valuable surgical option. This approach offers excellent exposure for accurate intraoperative assessment of invasion and resectability, while maintaining a relatively low invasiveness through sternoclavicular joint preservation and limited sternal division [5]. Importantly, selected cases may still be amenable to complete resection, even when preoperative imaging indicates extensive contact with surrounding organs or major vessels, as demonstrated here. Selecting an approach that enables optimal visualization and tactile assessment is crucial for intraoperative evaluation of resectability, rather than relying solely on imaging findings. Although magnetic resonance imaging may provide additional information regarding local invasion and resectability in mediastinal neurogenic tumours, it was not performed in the present case. Surgical planning was based on CT findings, and resectability was ultimately determined by intraoperative assessment. In mediastinal tumours surrounded by vital structures, the TMA provides superior exposure with less invasiveness and improved safety. Moreover, even when intraoperative findings reveal unresectability and necessitate aborting curative resection, this approach enables a safe and minimally disruptive transition to alternative treatments. To improve prognosis, meticulous preoperative planning—including careful surgical approach selection—along with accurate intraoperative assessment of resectability and active pursuit of complete resection whenever feasible, are crucial.

## Author Contributions

M.S. drafted the manuscript. M.S., S.N. and T.F.C.‐Y. performed the surgery. All authors contributed to manuscript discussion and revision. S.N. and T.F.C.‐Y. supervised manuscript editing. All authors approved the final manuscript.

## Funding

The authors have nothing to report.

## Ethics Statement

The authors declare that written informed consent was obtained for the publication of this manuscript and accompanying images and attest that the form used to obtain consent from the patient complies with the Journal requirements as outlined in the author guidelines.

## Conflicts of Interest

The authors declare no conflicts of interest.

## Data Availability

Data sharing not applicable to this article as no datasets were generated or analysed during the current study.
